# Case report: Continuous spinal cord physiologic monitoring following traumatic spinal cord injury—A report from the Winnipeg Intraspinal Pressure Study (WISP)

**DOI:** 10.3389/fneur.2023.1069623

**Published:** 2023-04-11

**Authors:** Perry Dhaliwal, Alwyn Gomez, Frederick Adam Zeiler

**Affiliations:** ^1^Section of Neurosurgery, Department of Surgery, Rady Faculty of Health Sciences, University of Manitoba, Winnipeg, MB, Canada; ^2^Department of Human Anatomy and Cell Science, Rady Faculty of Health Sciences, University of Manitoba, Winnipeg, MB, Canada; ^3^Biomedical Engineering, Faculty of Engineering, University of Manitoba, Winnipeg, MB, Canada; ^4^Department of Clinical Neuroscience, Karolinska Institute, Stockholm, Sweden; ^5^Centre on Aging, University of Manitoba, Winnipeg, MB, Canada; ^6^Division of Anaesthesia, Department of Medicine, Addenbrooke's Hospital, University of Cambridge, Cambridge, United Kingdom

**Keywords:** spinal cord injury, spinal cord perfusion pressure, intraspinal pressure, autoregulation, spinal cord

## Abstract

**Introduction:**

Acute traumatic spinal cord injury is routinely managed by surgical decompression and instrumentation of the spine. Guidelines also suggest elevating mean arterial pressure to 85 mmHg to mitigate secondary injury. However, the evidence for these recommendations remains very limited. There is now considerable interest in measuring spinal cord perfusion pressure by monitoring mean arterial pressure and intraspinal pressure. Here, we present our first institutional experience of using a strain gauge pressure transducer monitor to measure intraspinal pressure and subsequent derivation of spinal cord perfusion pressure.

**Case presentation:**

The patient presented to medical attention after a fall off of scaffolding. A trauma assessment was completed at a local emergency room. He did not have any motor strength or sensation to the lower extremities. A computed tomography (CT) scan of the thoracolumbar spine confirmed a T12 burst fracture with retropulsion of bone fragments into the spinal canal. He was taken to surgery for urgent decompression of the spinal cord and instrumentation of the spine. A subdural strain gauge pressure monitor was placed at the site of injury through a small dural incision. Mean arterial pressure and intraspinal pressure were then monitored for 5 days after surgery. Spinal cord perfusion pressure was derived. The procedure was performed without complication and the patient underwent rehabilitation for 3 months where he regained some motor and sensory function in his lower extremities.

**Conclusion:**

The first North American attempt at insertion of a strain gauge pressure monitor into the subdural space at the site of injury following acute traumatic spinal cord injury was performed successfully and without complication. Spinal cord perfusion pressure was derived successfully using this physiological monitoring. Further research efforts to validate this technique are required.

## Introduction

Traumatic spinal cord injury research in the clinical setting has focused on managing secondary injury by early decompression of the spinal cord and optimizing spinal cord perfusion. Expert guidelines (1) have recommended maintaining mean arterial pressure greater than 85 mmHg following acute spinal cord injury though the evidence for this practice is largely limited to retrospective studies. Over the past decade, researchers have employed various methods to measure perfusion of the spinal cord after traumatic spinal cord injury. Available methods of measuring spinal cord perfusion and autoregulation include the use of lumbar subarachnoid drains (2) and strain gauge pressure transducers placed at the site of injury (3). This is the first North American report of a single center observational study designed to validate the methodology of invasive intraspinal pressure monitoring for patients with traumatic spinal cord injury (ASIA A, B or C) (4). The study was approved by the local research ethics board at the University of Manitoba and institutional review board.

## Case

**Figure 1:**
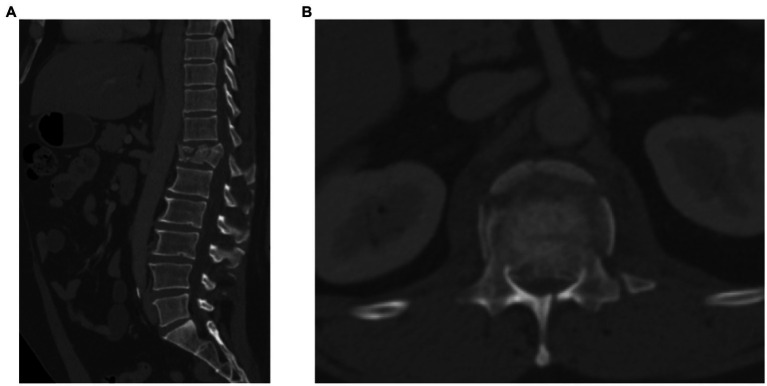
CT scan images of thoracolumbar spine demonstrating spinal fracture. **(A)** Sagittal CT scan showing T12 fracture with comminuted vertebral body. **(B)** Axial CT scan at T12 level demonstrating violation of the posterior cortex of vertebral body with retropulsion of bone fragments.

The patient presented to medical attention after a fall off of scaffolding at a work site. Upon arrival, the patient was assessed by the trauma team and was found to be hemodynamically stable. His airway was patent and he did not require endotracheal intubation. Neurological examination revealed that he had normal cranial nerve function. He had normal strength in all myotomes of the upper extremities. He had normal sensation in all dermatomes of the upper extremities. Examination of the lower extremities revealed 3/5 strength in the hip flexors and 0/5 strength in knee extension plantar flexion or dorsiflexion based on the Medical Research Council Manual Muscle Testing Scale. There was patchy sensation to light touch and pin-prick in a non-dermatomal pattern in the lower extremities. He had no rectal tone, voluntary anal contraction or deep anal pressure. He had complete loss of sensation in the perineal region bilaterally. The patient did not have reflexes at the patellar tendon or Achilles tendon. A complete trauma assessment was performed and CT scan of the lumbar spine revealed a severely comminuted T12 burst fracture with retropulsion of bone fragments into the spinal canal (see Figure 1). No other traumatic injuries were found. We attempted to arrange for an urgent MRI scan of the spine but were not able to arrange this in a timely fashion due to resource limitations at our institution during the coronavirus pandemic. The patient was diagnosed with a complete spinal cord injury. Surgical intervention was recommended and the patient was consented for decompression and stabilization. The time course of events is described in detail in Table 1.

**Table 1:** Time course of events for examination, imaging and intervention on a patient with an acute traumatic spinal cord injury.

Day	Time	Description
1	9:24 am	Patient presents to the tertiary care hospital for assessment after a fall off of scaffolding.
	9:53 am	Trauma assessment is completed by the emergency room physician.
	9:57 am	CT scan of the brain, chest, abdomen, pelvis and spine are completed.
	10:20 am	Spine surgery consultation. History is obtained and detailed neurological examination is performed. The patient is confirmed to have 3/5 strength to the hip flexors and 0/5 strength in the remaining muscle groups of the lower extremities. He had patchy sensation to light touch and pin prick stimulation in a non-dermatomal fashion over both lower extremities. Digital rectal examination demonstrated absence of rectal tone, voluntary anal contraction and deep anal pressure sensation. He also had complete loss of sensation bilaterally in the perineal region. Imaging confirms a T12 burst fracture with canal compromise. The patient is diagnosed with a complete spinal cord injury. Surgical intervention for decompression and stabilization are recommended.
	1:00 pm	The patient undergoes surgical intervention in the form of a T10-L2 posterior instrumented fusion with T12 and L1 laminectomy. A subdural strain gauge pressure monitor is inserted at the T12 level.
Day 2–6	-	The patient is monitored in an intermediate care unit following surgery for a period of 5 days. Physiologic data including arterial blood pressure, heart rate and intraspinal pressure are captured using invasive monitoring.
Day 7	-	The subdural catheter and arterial line are removed at the bedside. End of physiological monitoring.
Day 17	-	The patient is transferred to the spinal cord injury rehabilitation unit.
6 Months post-surgery	-	Outpatient follow up with patient.

### Surgical intervention

The patient was brought to the operating room in an emergent fashion. He was taken to surgery within 3 h of presentation to hospital and within 4 h of his injury. The patient underwent endotracheal intubation and a general anesthetic was administered. An arterial line and foley catheter was placed. Sequential compression devices are placed on the lower extremities. The patient was then flipped onto the operative table using standard spine precautions. His head was positioned in a Dupaco frame and the extremities were padded to prevent peripheral nerve compression. The thoracolumbar area was cleansed with chlorhexidine solution and operative drapes were applied. The patient received preoperative antibiotics and the anesthesia team was instructed to maintain a mean arterial pressure (MAP) of 85 mmHg throughout the operative intervention. Corticosteroids were not administered in the preoperative, intra-operative or postoperative phases of management of this patient. Intra-operative x-rays were used to confirm the surgical level. Thereafter, a midline incision was made with a scalpel and cautery was used to perform a dissection to expose the thoracolumbar region from the T10 to L2 vertebral levels. A second set of intra-operative x-rays were obtained following initial exposure to verify the surgical levels. Once confirmed the exposure was optimized and deeper retractors were placed. Pedicle screws were placed using standard free-hand techniques at T10, T11, L1, and L2. A laminectomy was then performed at the T12 and L1 levels by removal of the spinous process and drilling of the lamina. The inferior half of T11 was also removed. Kerrison rongeurs were used to complete the decompression. Thereafter, the superior and inferior facets of T12 were removed on the left side thereby isolating the T12 pedicle. The T12 pedicle was drilled away and the T12 nerve root on the left-hand side could be seen. A suture ligature was applied to the T12 nerve root and the nerve root was sectioned. This allowed for exposure ventral to the dura and visualization of the fracture fragments. A curette was used to push the fracture fragments back into the vertebral body and an ultrasound was used to confirm the extent of decompression. Once satisfied with the decompression, titanium rods were placed across the screw heads and were secured in place. The bone was then decorticated from T10 to L2 and a combination of locally harvested autograft and allograft was placed in the posterolateral space to create a fusion bed.

**Figure 2:**
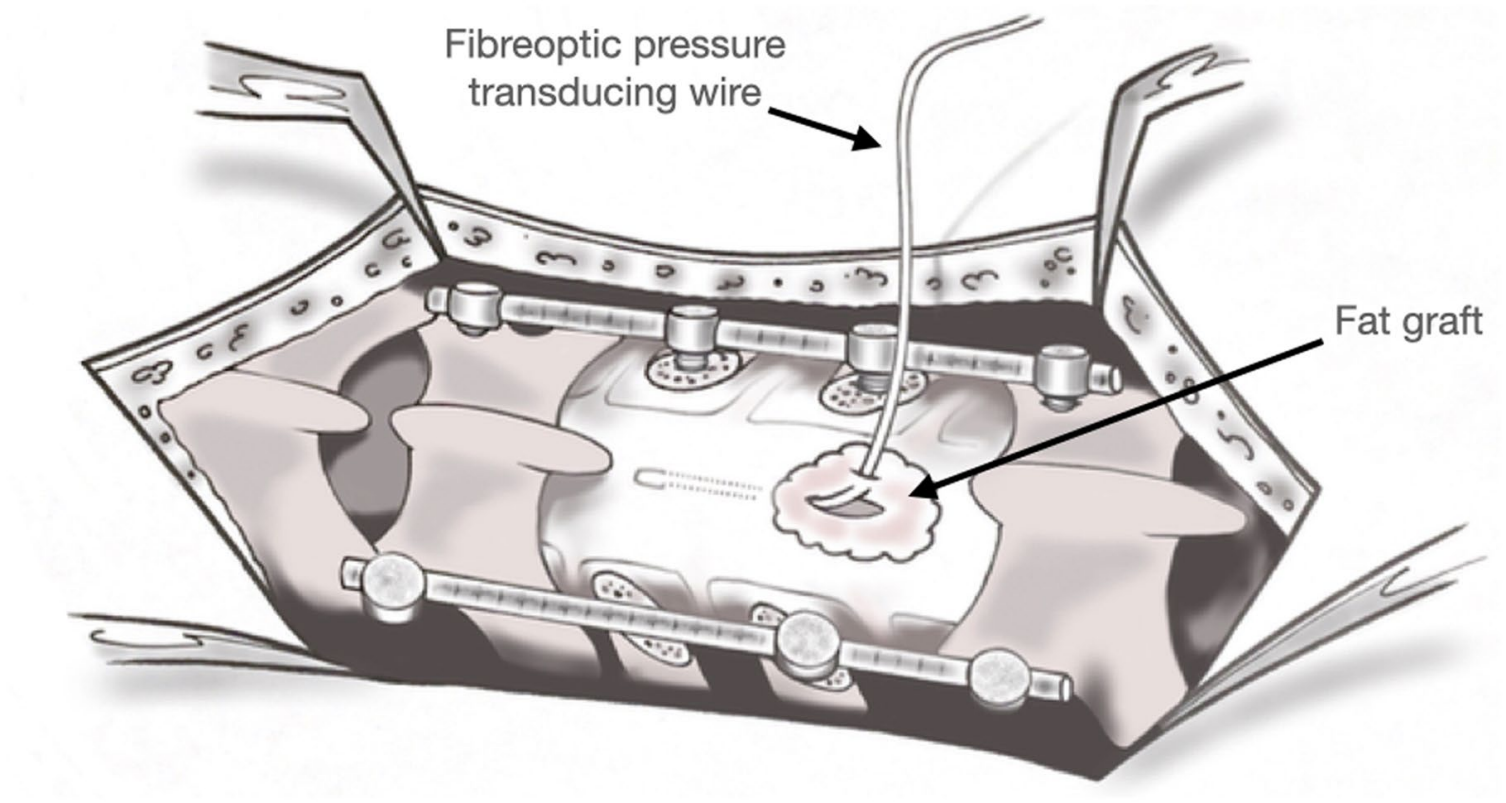
Graphical representation of the surgical site. The fiberoptic wire is seen placed through the dura into the subdural space at the site of injury. A fat graft was placed at the site of insertion to prevent pseudomeningocele formation.

Following the stabilization and decompression, we prepared for placement of the strain gauge pressure monitoring device (Codman ICP MicroSensor; Codman & Shurtleff Inc., Raynham, MA). The wire was first calibrated as per the manufacturer’s instructions and was prepared for insertion by passing it through the skin and into the surgical bed. A 6–0 gortex suture was used to apply tension to the dura at the level of L1 and a scalpel was used to make a small durotomy. While, ensuring the dura remained under gentle tension, the strain gauge wire was placed into the subdural space with minimal loss of CSF. An intra-operative ultrasound was then used to visualize the intradural space and the wire was positioned adjacent to the area of spinal cord injury at the T12 level. The strain gauge wire could not be visualized clearly when held still, but the position of the catheter could be ascertained as it was moved in a rostro-caudal fashion. Once satisfied with the position, the surgical assistant held it in place as the primary surgeon placed a purse string suture with the 6–0 gortex suture around the site of dural entry to hold the catheter in position and to close the dura around the catheter itself. A locally harvested fat graft was then placed over the site where the catheter was entering the dural opening (see Figure 2). The fat graft was then coated in fibrin glue to hold it in place (Tisseel Fibrin Sealant, Baxter Healthcare Corporation, Deerfield, IL, USA). The wound was then closed in standard fashion and the catheter was taped to the patient to prevent it from being dislodged. The patient was then transferred to an intermediate care unit for ongoing physiological monitoring. Physiological data was captured for the first 5 days following surgery and then the subdural catheter was removed at the bedside. The catheter removal was performed by removal of the adhesives and by simply pulling the catheter using manual traction from the skin insertion point. This was performed without difficulty. The catheter was inspected to ensure complete removal. The skin insertion site was then closed with a single suture at the bedside to prevent any postoperative cerebrospinal fluid leakage.

### Physiological monitoring

#### Physiologic data acquisition

While in the intermediate care unit, physiological parameters such as heart rate, arterial blood pressure (ABP) and intraspinal pressure (ISP) were captured at the bedside. Arterial line was zeroed at the level of the right atrium, using radial arterial lines. ISP was monitored using intra-thecal Codman strain-gauge pressure sensors, through the above-described methodologies (3, 5, 6). ABP and ISP were monitored using invasive methods with all signals recorded in high frequency time series, sampled at 100 Hz or higher through analog to digital signal converters (DT9804 or 9 T9826; Data Translations, Marlboro, MA), using ICM+ software (Cambridge Enterprise Ltd., Cambridge, UK, http://www.neurosurg.cam.ac.uk/icmplus) connected to our bedside monitors (7, 8). Signals from all of the monitoring devices described below were subsequently recorded in time series using this software over the course of the recording periods described above. This is similar to acute brain injury data collection methodologies previously published by our group (9–13). We did not attempt to manipulate intraspinal pressure or mean arterial pressure during the recording period.

#### Data processing

Post-acquisition processing of the above signals was be conducted using ICM+ software. Spinal cord perfusion pressure (ScPP) was calculated using the formula: SCPP = MAP – ISP. Ten second moving averages (updated every 10 s to avoid data overlap) were be calculated for all recorded signals: ISP, ABP (which produced MAP), and ScPP. Ten second moving averages were be calculated in order to focus on slow-waves of parent signals, decimating the frequency to the range associated with vascular autoregulation, similar to the brain injury literature (14, 15).

Spinal vascular reactivity (ScPRx) indices were derived using a moving Pearson correlation coefficient calculated between ISP and MAP using 30 consecutive 10 s windows (i.e., 5 min of data), updated every minute. This was conducted in accordance with previous ScPRx work, adapting methodologies from the moderate/severe brain injury literature (3, 5, 16, 17). Similarly, optimal spinal cord perfusion pressure (ScPPopt) was derived, adapting current multi-window weighted approaches utilized for optimal cerebral perfusion pressure (CPPopt) derivation in acute brain injury (18–21).

#### Descriptive physiologic findings

**Figure 3:**
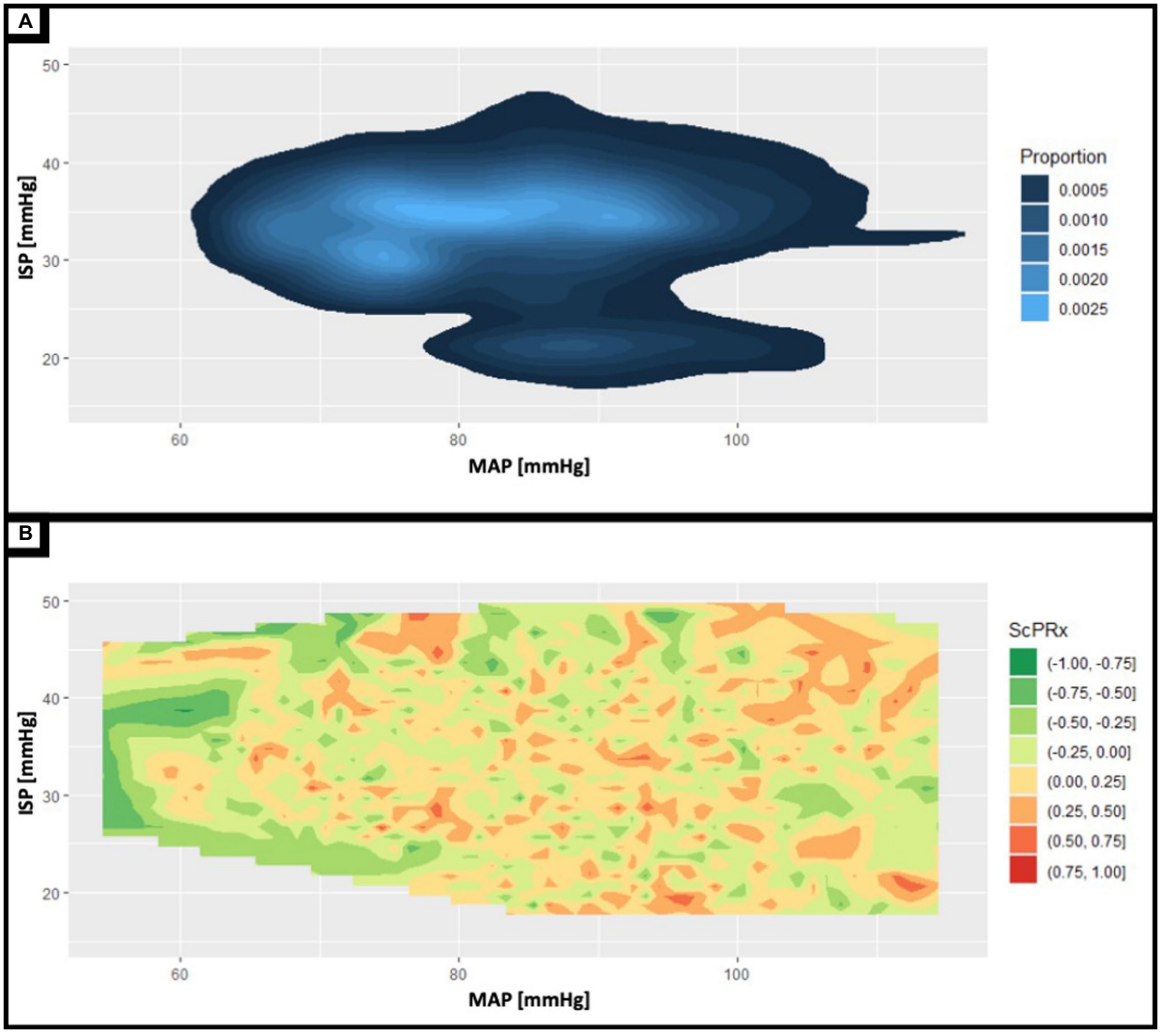
**(A)** Shows a two-dimensional contour plot of the patient’s entire recording period displaying the time spent at various mean arterial pressures (MAP) and intraspinal pressures (ISP). The lighter the shade of blue the greater proportion of time spent at the corresponding MAP and ISP. Notably, there is a peninsula in with lower ISP values attributable to the immediately postoperative period with ISP values ranging from approximately 15-25 mmHg. **(B)** Shows a contour plot examining the relationships between ISP, MAP, and spinal cord pressure reactivity index (ScPRx). ScPRx ranges in color from red (most disrupted) to green (most intact). There seems to be a trend with low concurrent ISP and MAP being associated with intact ScPRx while high ISP and MAP is associated with disrupted ScPRx.

Figure 3 displays data density (Panel A) and contour plots for MAP, ISP and ScPRx. Panel A demonstrates data distribution for paired measures of MAP and ISP. Note the high pressure values for ISP, as the patient was prone with various levels of incline to the head of the bed during their recordings. Panel B highlights preliminary data into the complex relationship between MAP, ISP, and ScPRx. In the traumatic brain injury literature, a pressure reactivity index greater than +0.4 to +1.0 reflects abnormal cerebral autoregulation such that the mean arterial pressure results in direct passive changes in cerebral flood flow (22). Here, we present a preliminary analysis demonstrating that the spinal vascular reactivity index (ScPRx) trends towards abnormal ranges where the intraspinal pressure and mean arterial pressure are high. Such analysis remains exploratory and is provided as only an example of the novel physiologic insights that can be explored with this type of spinal physiologic information.

**Figure 4:**
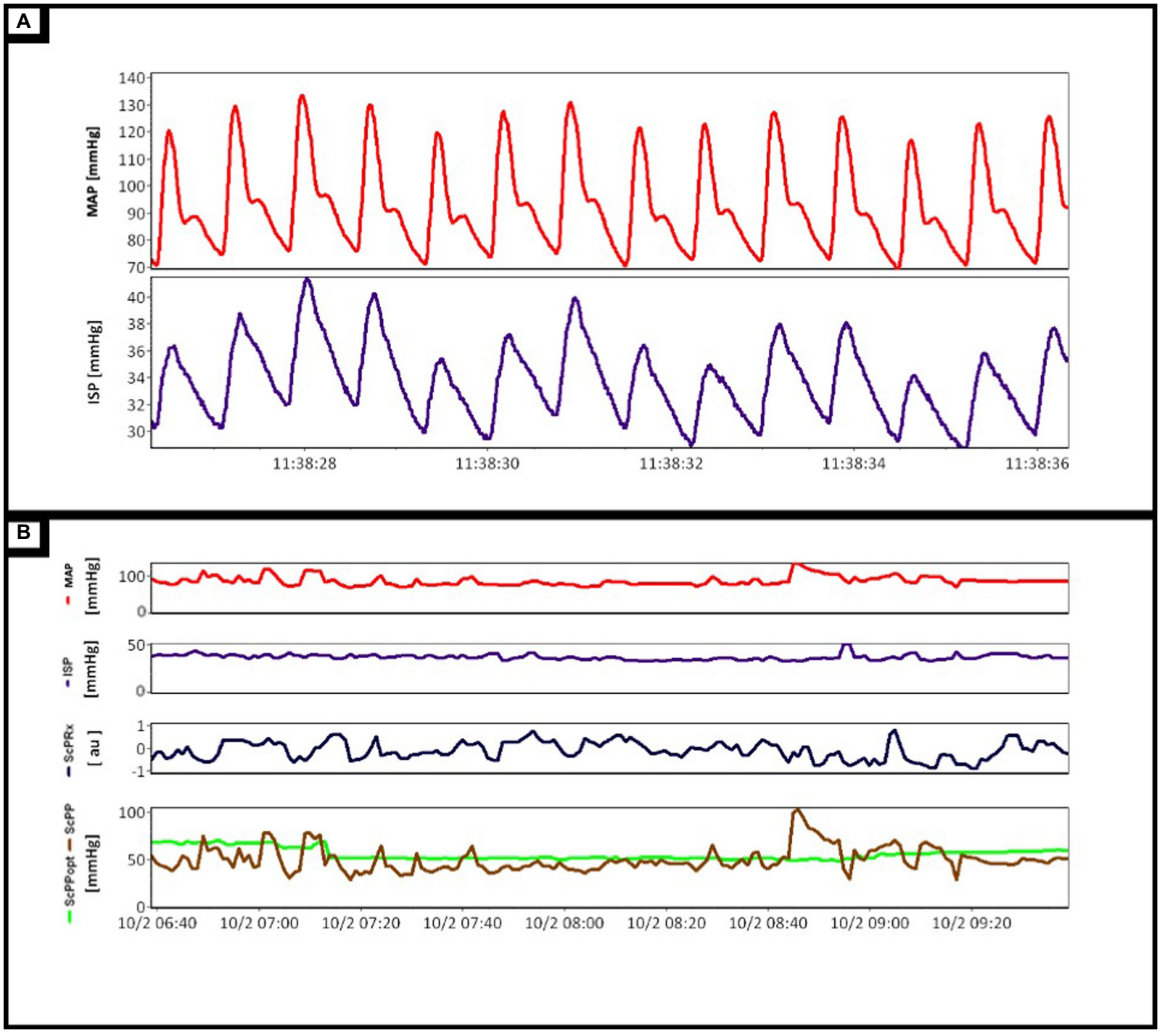
**(A)** Demonstrated the mean arterial pressure (MAP, red line) and intraspinal pressure (ISP, purple line) waveforms as they are recorded in real-time. **(B)** Displays the processed minute-to-minute physiologic data. This includes directly measured parameters such as MAP (red line) and ISP (purple line) as well as derived parameters, spinal cord pressure reactivity index (ScPRx, black line), spinal cord perfusion pressure (ScPP, brown line), and optimal spinal cord perfusion pressure (ScPPopt, green line).

In Figure 4, Panel A displays examples of the continuous full waveform (100 Hz) time trends of both ABP and ISP. Of note, the recorded ISP displays a robust pulsatile waveform, consistent with the cardiac cycles and existing invasive intracranial pressure data. This highlights the feasibility of the described techniques for accurate high-frequency data capture in this patient cohort. Similarly, Figure 4 Panel B highlights post-processed intraspinal physiologic data (minute update frequency), demonstrating continuously updating MAP, ISP, ScPRx, ScPP, and ScPPopt. Similar to the brain injury literature, this preliminary analysis highlights signal variability in ISP and ScPRx, similar to that seen for ICP and PRx. Further, the ability to derive ScPPopt, using ScPP and ScPRx, has been displayed here, though interpretation of such metrics is unclear at this time due to lack of literature.

### Follow up

The patient was assessed 6 months following injury. After 2 months in rehabilitation, functional improvements were noted with functional independence measure (FIM) improving from 70/126 to 116/126. Similarly, the spinal cord independence measure (SCIM-III) improved from 48/100 to 67/100. The patient was able to ambulate with ankle-foot orthoses and a walker for approximately 150 ft. though he was dependent on a wheelchair for longer distances. The patient did not regain control of bowel or bladder function. No surgical complications were noted and the patient did not develop a pseudomeningocele.

## Discussion

The Winnipeg Intraspinal Pressure Study (WISP) is a study designed to validate the technique of intraspinal pressure monitoring with the use of a strain gauge pressure monitoring wire (4). Here, we present the first attempt at measurement of intraspinal pressure through the use this technique at our center. To our knowledge, this is the first North American report on the use of this technique. Through this attempt, we have demonstrated that (a) intraspinal pressure can be measured from the site of injury, (b) the intraspinal pressure can be used to derive values for spinal cord perfusion pressure and (c) that the technique can be performed safely. Thus, this case represents an important step forward in understanding spinal cord pathophysiology following acute traumatic spinal cord injury in humans.

The application of strain gauge pressure monitoring devices after acute traumatic spinal cord injury was first reported by Werndle et al. (16). The comprehensive body of work that followed demonstrated that the intraspinal pressure waveform could be characterized in relation to a patient’s heart rate, respiratory rate and blood pressure. The intraspinal pressures were also compared to pressures obtained from the epidural space and from catheters placed in the spinal cord parenchyma (5). Spinal cord perfusion pressure was derived by calculating the difference between the mean arterial pressure and intraspinal pressure (17). Pearson correlation coefficients were used to derive measures of spinal cord autoregulation and compliance such as the reactivity index (ScPRx) (23). Various manipulations of blood pressure were also applied to assess the impact on spinal cord perfusion pressure (16). In this case report, we saw trends towards higher values of ScPrx when intraspinal pressure and mean arterial pressure were elevated while lower ISP and MAP were correlated with ScPRx values in lower ranges.

Despite the extensive physiologic data obtained from this technique, others have indicated concerns related to the possibility of cerebrospinal fluid leakage around the insertion of the wire and the possibility of exacerbating neurological injury through placement of the wire at the site of injury (24). Moreover, it remains unclear where the catheter should be positioned in relation to the site of injury. During our first attempt, we attempted to mitigate some of these concerns through the use of ultrasound guidance to place the strain gauge wire in the center of the site of injury. We also placed a fat graft obtained from the patient’s incision around the dural incision to reduce the risk of a post-operative pseudomeningocele. These techniques will need to be further refined and implemented on more patients to verify whether they enhance the safety of this method of intraspinal pressure monitoring.

Another method of measuring spinal cord perfusion pressure involves the use of a lumbar subarachnoid drain (2). Lumbar subarachnoid drains have been used during aortic aneurysm surgery to improve spinal cord perfusion by removing cerebral spinal fluid so as to maximize the differential pressure gradient between mean arterial pressure and intraspinal pressure thereby mitigating the risk of a spinal cord infarct (25). In the setting of traumatic spinal cord injury, measurement of intraspinal pressure through the use of a lumbar drain has allowed for derivation of spinal cord perfusion (26). However, it is notable that a comparative study did not find correlations between spinal cord perfusion pressure derived from lumbar CSF drainage compared to values derived from the use of a strain gauge wire placed at the site of injury (27). Though questions remain about the validity of this technique, some have adopted lumbar CSF drainage as part of a routine protocol in the management of traumatic spinal cord injury (28).

The use of invasive monitoring techniques to monitor spinal cord physiology following spinal cord injury will have important implications for the management of these patients. Despite these early successes, future research efforts will need to further characterize cerebrospinal fluid dynamics in relation to traumatic spinal cord injury. The use of a strain gauge wire to measure intraspinal pressure needs to be further validated through larger studies. Additional avenues for research should be aimed at assessing the impact of blood pressure manipulation on spinal cord perfusion pressure and functional outcomes. Correlation between spinal cord autoregulation and spinal cord metabolism and markers of neuronal injury may also provide useful information to guide targeted therapies for spinal cord injury.

## Conclusion

The first North American attempt at insertion of a strain gauge pressure monitor into the subdural space at the site of injury following acute traumatic spinal cord injury was performed successfully and without complication. Intraspinal pressure was measured and spinal cord perfusion pressure was derived successfully using this physiological monitoring. Further research efforts to validate this technique are required.

## Data availability statement

The raw data supporting the conclusions of this article will be made available by the authors, without undue reservation.

## Ethics statement

The study was approved by the local research ethics board at the University of Manitoba and institutional review board. The patients/participants provided their written informed consent to participate in this study. Written informed consent was obtained from the participant/patient(s) for the publication of this case report.

## Author contributions

PD drafted the manuscript. AG designed the figures for the study and analyzed the data. FZ edited drafts of the manuscript and analyzed data. All authors contributed to the article and approved the submitted version.

## Funding

PD’s research is supported by the Thorlakson Chair in Surgical Research Establishment Award through the Department of Surgery at the University of Manitoba. Research funding was also received from the Health Sciences Centre Foundation and the Praxis Spinal Cord Institute. FZ’s contribution to this work was directly supported through the Natural Sciences and Engineering Research Council of Canada (NSERC) (DGECR-2022-00260, RGPIN-2022-03621, and ALLRP-576386-22) and the Manitoba Public Insurance (MPI) Neuroscience Research Operating Fund. Acknowledgements: FZ receives research support from NSERC, CIHR, the MPI Neuroscience Research Operating Fund, the Health Sciences Centre Foundation Winnipeg, the Canada Foundation for Innovation (CFI) (Project #: 38583), Research Manitoba (Grant #: 3906), the University of Manitoba VPRI Research Investment Fund (RIF), and the University of Manitoba MPI Professorship in Neuroscience. AG is supported through the University of Manitoba Clinician Investigator Program, the University of Manitoba Dean’s Fellowship, the Manitoba Medical Services Foundation Research and Education Fellowship, the R. Samuel McLaughlin Research Fellowship, and a Canadian Institutes of Health Research (CIHR) Fellowship (Grant #: 472286).

## Conflict of interest

The authors declare that the research was conducted in the absence of any commercial or financial relationships that could be construed as a potential conflict of interest.

## Publisher’s note

All claims expressed in this article are solely those of the authors and do not necessarily represent those of their affiliated organizations, or those of the publisher, the editors and the reviewers. Any product that may be evaluated in this article, or claim that may be made by its manufacturer, is not guaranteed or endorsed by the publisher.
